# The rate and associated risk factors for acute carpal tunnel syndrome complicating a fracture of the distal radius

**DOI:** 10.1007/s00590-021-02975-5

**Published:** 2021-04-23

**Authors:** Jun Min Leow, Nicholas D. Clement, Margaret M. McQueen, Andrew D. Duckworth

**Affiliations:** Edinburgh Orthopaedics–Edinburgh Orthopaedic Trauma and the University of Edinburgh, Royal Infirmary of Edinburgh, Edinburgh, EH16 4SA UK

**Keywords:** Acute carpal tunnel syndrome, Distal radius fractures, Diagnosis, Management

## Abstract

**Background:**

Acute carpal tunnel syndrome (ACTS) is a known complication of distal radius fractures. This study aimed to document the demographics, range of presenting symptoms and risk factors of patients who develop ACTS following a fracture of the distal radius.

**Methods:**

A retrospective review of 1189 patients with an acute distal radius fracture treated in the study centre over a one-year period were identified. Demographic and clinical variables were collected and compared between controls (did not develop ACTS) and those patients who did develop ACTS to identify factors associated with developing ACS.

**Results:**

There were 51 (4.3%) distal radius fractures complicated by ACTS. The mean age of patients who developed ACTS was 56 years (range, 16–89) and 73% were female. The median time of onset post-injury was one week (range, 1–12). There was no association between patient background and comorbidities with the development of ACTS. AO-OTA Type C fractures had significantly increased rates of developing ACTS.

**Conclusion:**

Four percent of distal radius fractures were complicated by ACTS. There was no association between patient background and comorbidities with the development of ACTS. AO-OTA type C complete articular fractures had a significantly higher rate of ACTS. A suggested treatment algorithm for patients presenting with ACTS has been presented.

**Level of evidence:**

III.

## Introduction

Acute carpal tunnel syndrome (ACTS) is a recognised complication following a fracture of the distal radius and is due to elevated compartment pressure within the carpal tunnel [1–3]. ACTS associated with a distal radial fracture has a reported incidence of between 3 and 17% [4–9] and could result in permanent median nerve dysfunction or complex regional pain syndrome (CRPS) if left unrecognised or untreated [10–12]. Although the condition can be treated with surgical decompression of the carpal tunnel, the diagnosis of ACTS and subsequent selection of patients to be treated surgically can be challenging [13]. Symptoms are often non-specific, which leads to a wide range in the reported incidence of ACTS following distal radius fractures [4–9].

The Carpal Tunnel Syndrome-6 (CTS-6) is a six-item clinical diagnostic algorithm that takes into consideration two symptoms (paraesthesia in the median nerve distribution and nocturnal symptoms), two signs of advanced disease (atrophy of thenar muscles and reduced 2-point discrimination) and two positive provocation tests (Tinel’s test and Phalen’s test) [14]. However, it is intended for cases of chronic carpal tunnel syndrome, as the provocative tests do not exhibit good sensitivity and are difficult to perform in ACTS. Clinical diagnostic algorithms can aid in stratifying the probability of carpal tunnel syndrome rather than in making a definitive diagnosis [15].

There is a paucity of evidence that defines the epidemiology, presenting signs and symptoms and management of patients presenting with ACTS following a distal radius fracture. Studies have shown that AO Type-C fractures, open fractures, fracture translation and diabetes mellitus are risk factors for developing ACTS [16, 17]. However, these studies investigated patients who developed ACTS following open reduction internal fixation (ORIF), which may represent a confounding factor for development of ACTS [18, 19]. Distal radius fractures are one of the commonest types of fractures and a majority of them can be treated conservatively [20–22]. Therefore, knowledge of the epidemiology and risk factors associated with developing ACTS from a general fracture population could help surgeons in the diagnosis and subsequent planning of management.

The primary aim of this study was to describe the prevalence and demographics of patients that developed ACTS in association with a fracture of the distal radius from a defined population. The secondary aim was to determine risk factors associated with development of ACTS following a distal radius fracture.

## Methods

A consecutive series of 1189 patients with an acute fracture of the distal radius who presented to the study centre over a period of 12 months were recorded. The study was reviewed by the local NHS Research Ethics Service (NR/1411AB8) and was registered with the local musculoskeletal quality improvement committee.

### Demographics

Demographic details including age, gender, co-morbidities, Scottish Index of Multiple Deprivation (SIMD) [23–25], smoking status, weekly alcohol consumption in units, mechanism of injury, fracture classification, and definitive fracture management were recorded prospectively. Patients under the age of 16 years were excluded.

### Diagnosis of ACTS

CTS was defined as paraesthesia affecting any of the radial three and a half fingers within 12 weeks from the date of injury [1–3]. Patients developing symptoms within 1 week were included in the acute-onset group, and patients developing symptoms from 1 to 12 weeks were defined as the subacute-onset group [26]. Nocturnal symptoms, wasting of the thenar eminence and positive provocation tests were recorded but not included in the diagnostic criteria. Patients with a history of chronic carpal tunnel syndrome, previous carpal tunnel decompression, symptoms arising after 12 weeks from time of injury, and iatrogenic causes of carpal tunnel syndrome were excluded.

### Radiographs

Standard postero-anterior and lateral radiographs of the wrist taken at the time of injury were prospectively classified by two orthopaedic surgeons (ADD, NDC) using the AO-OTA classification system [27].

### Management

Patients presenting to the Emergency Department (ED) in the study centre with a displaced distal radius fracture with ACTS would undergo a manipulation under sedation or intravenous regional anaesthesia (MUA). Definitive management of ACTS was determined by a consultant orthopaedic trauma surgeon. If ACTS persisted or evolved in the presence of an unstable fracture pattern, patients would undergo surgical stabilisation (ORIF or non-bridging external fixation) with concomitant carpal tunnel decompression (CTD). For patients who developed ACTS between two to six weeks, conservative management was used and the evolution of symptoms monitored closely, with definitive fixation and/or CTD considered if fracture displacement was thought to be the cause. For patients who developed ACTS after 6 weeks, treatment options included conservative measures (e.g. night splint), CTD, or a distal radius osteotomy with CTD.

### Follow-up protocol

The minimum period for clinical follow up was six weeks and all electronic patient records were reviewed at a minimum of three years following injury. Patients progressing with no complications were routinely discharged at six weeks, with physiotherapy if indicated. Patients were advised to contact the study centre if they have any new or worsening problems. The study centre is the only health board for the catchment population thus any further complications or representations should have been identified.

### Statistical methods

SPSS Version 18.0 (SPSS, Chicago, Illinois) was used for statistical analysis. Demographic and clinical variables were then compared with controls to determine their correlation with CTS development. Fisher’s exact test and Chi-squared test was used for categorical data. Student’s *t* test was used for continuous data. Two-tailed *p*-values were reported and statistical significance was set at a *p* value < 0.05, with 95% confidence intervals (CI) presented.

## Results

**Fig. 1:**
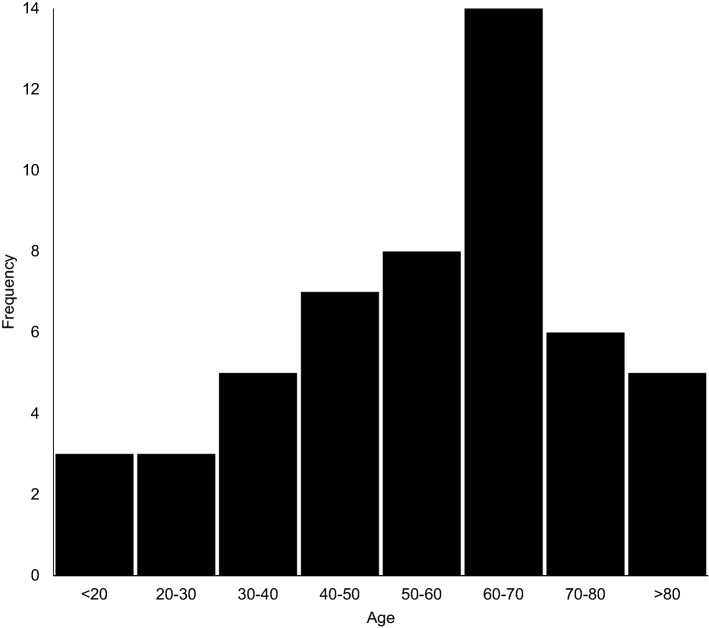
Histogram showing the frequency in which carpal tunnel syndrome presents in different age groups and gender

There were 51(4.3%) patients in the study cohort of 1189 patients with a distal radius fracture who developed ACTS. The mean age of these patients was 56 years (range 16-89 years; SD 18.9) and the most common age group to present with ACTS was 60–70 years (*n* = 14/52, 27%; Fig. 1). The majority (*n* = 36,73%) of ACTS cases were female (Table 1). The median time of onset post-injury was one week (range, 1–12 weeks). The most commonly sustained fracture among the CTS group were type-A extra-articular fractures (*n* = 26, 51%), followed by type-C complete articular fractures (*n* = 22, 43%) and finally type-B partially articular fractures (*n* = 3, 6%).

**Table 1:** Risk factors for carpal tunnel syndrome

	No CTS *n* (%)	CTS *n* (%)	*p* value
Total	1138 (95.7)	51 (4.3)	NA
*Gender*			
Male	305 (26.8)	15 (29.4)	0.681*
Female	833 (73.2)	36 (70.6)	
Mean age (range, SD, 95% CI)	59 (16–100, 20.8, 57–60)	56 (16–89, 18.9, 50–61)	0.315^a^
*Mechanism of injury*			
Fall from standing height (*n* = 963)	923 (81.1)	40 (78.4)	0.841*
Fall from height (*n* = 58)	53 (4.7)	5 (9.8)	
Sports (*n* = 108)	102 (9.0)	4 (7.8)	
MVC (*n* = 41)	40 (3.5)	1 (2.0)	
Crush (*n* = 4)	4 (0.4)	0 (0)	
Fight/assault (*n* = 11)	10 (0.9)	1 (2.0)	
Direct blow (*n* = 2)	2 (0.2)	0 (0)	
Other (*n* = 3)	3 (0.3)	0 (0)	
Missing (*n* = 1)			
*Co-morbidities* ≥ *1*	583 (51.2)	21 (41.2)	0.160*
Diabetes	52 (4.6)	0 (0)	0.164
Thyroid disease	31 (2.7)	4 (7.8)	0.059
Pregnancy	0 (0)	1 (2.0)	**0.043**^¶^
BMI (range, SD, 95% CI)	25 (11.4–44.0, 4.6, 25–26) (*n* = 872)	26 (18.0–38.5, 3.9, 24–27) (*n* = 42)	0.521^a^
Smokers (*n* = 944)	182 (20.1)	8 (20.5)	0.951*
Alcohol excess (*n* = 953)	63 (6.9)	2 (4.8)	1.000
*Index of multiple deprivation*			
1 (*n* = 141)	136 (13.5)	5 (11.1)	0.840*
2 (*n* = 194)	188 (18.6)	6 (13.3)	
3 (*n* = 146)	140 (13.9)	6 (13.3)	
4 (*n* = 194)	185 (18.3)	9 (20.0)	
5 (*n* = 379)	360 (35.7)	19 (42.2)	
Missing (*n* = 135)			
*Occupation*			
Heavy manual (*n* = 28)	24 (2.2)	4 (8.3)	0.109*
Light manual (*n* = 145)	139 (13.0)	6 (12.5)	
Office work (*n* = 261)	249 (23.3)	12 (25.0)	
Retired (*n* = 555)	535 (50.1)	20 (41.7)	
Unemployed (*n* = 126	120 (11.2)	6 (12.5)	
Missing (*n* = 74)			
*AO-OTA*			
A (*n* = 757)	731 (64.2)	26 (51.0)	0.001*
B (*n* = 166)	163 (14.3)	3 (5.9)	
C (*n* = 266)	244 (21.4)	22 (43.1)	

*Chi-squared

^¶^Fisher’s exact test

^a^Student’s * t* test

### Presentation and management

All 51 patients had paraesthesia of at least one finger in the median nerve distribution from the time of injury up to 12 weeks post-injury (Table 2). Pain out with the proportion of the injury was seen in seven (13.7%) patients and five (9.8%) patients had reduced power. Two patients had wasting of the thenar eminence and presented after five weeks.

**Table 2:** Symptoms and signs of acute carpal tunnel syndrome

Description	*N* (%)
*Sensory symptoms*	
Paraesthesia	51 (100)
Pain	7 (13.7)
Cold	1 (2.0)
Nocturnal symptoms	3 (5.9)
*Motor signs and symptoms*	
Thenar muscle wasting	3 (5.9)
Motor weakness	6 (11.8)
Loss of function	1 (2.0)
Bilateral symptoms	1 (2.0)

Thirty-two patients (63%) developed ACTS within the first week. The definitive management is summarised in Table 3. Fifteen (47%) patients were treated with fracture fixation of which seven patients had a concomitant carpal tunnel decompression during their primary surgery. CTD was not carried out in cases where fracture displacement was thought to be the main cause of ACTS or where a patient had undergone an initial MUA with CTS symptom resolution but ultimately required fixation for fracture re-displacement. One patient had a late corrective osteotomy after failed non-operative management at ten months and another patient had a corrective osteotomy and carpal tunnel decompression at two months post-injury. Five (10%) patients from this group developed CRPS. Two of these patients had a corrective osteotomy with plate fixation and subsequently improved and the remaining three patients improved with physiotherapy alone.

**Table 3:** Treatment of carpal tunnel syndrome

Treatment	Acute (< 1 week) *N* (%)	Subacute (2–12 weeks) *N* (%)
Conservative	3 (9%)	3 (16%)
Wrist splint	0	4 (21%)
Steroid injection	0	1 (5%)
MUA	11 (34%)	0
Carpal tunnel decompression	1 (3%)	7 (37%)
Osteotomy (+ CTD)	2 (1 CTD) (6%)	2 (2 CTD) (11%)
Fracture fixation (+ CTD)	15 (7 CTD*) (47%)	2 (1 CTD) (11%)

*Of the eight patients who did not undergo concomitant CTD, six underwent non-bridging external fixation and two of these patients had an initial MUA that resolved their CTS symptoms but subsequently has displacement of their fracture and proceeded to ORIF

Nineteen (38%) patients developed ACTS one week after injury. The definitive management is summarised in Table 3. One patient had a non-bridging external fixator to correct dorsal displacement two weeks post-injury. Seven patients were treated with CTD alone, one patient had an ORIF and CTD, and two patients had an osteotomy and CTD. From our cohort, 20% (10 of 51) of patients who were treated without any intervention went on to resolve spontaneously.

### Risk factors

Age, sex, mechanism of injury, BMI, smoking status, history of alcohol excess, deprivation and occupation were not associated with ACTS (Table 1). Thyroid disease (*n* = 4) demonstrated a trend towards the development of ACTS (*p* = 0.059). ACTS was significantly associated with AO Type-C fractures (8.3%) compared to AO Type-A or Type-B fractures (3.1%) (*p* = 0.001). Patients with type C fractures had a higher chance of developing ACTS compared to those with type-A (odds ratio 2.76, 95% CI 1.53–4.55, *p* = 0.0004) or type-B (odds ratio 5.34, 95% CI 1.57–18.13, *p* = 0.003) fractures. Patients with AO type-C fractures who developed ACTS were treated with fracture fixation and carpal tunnel decompression (*n* = 8, 36%), fracture fixation alone (*n* = 4, 18%), CTD alone (*n* = 3, 14%), MUA (*n* = 3, 14%), wrist splint (*n* = 1, 4%) and conservatively (*n* = 3, 14%).

## Discussion

This study has defined the epidemiology, symptoms and signs, and management of patients presenting with ACTS complicating a fracture of the distal radius. The classical predisposing factors for idiopathic CTS were not associated with the development of ACTS. AO-OTA Type-C fractures was the most important risk factor associated with development of ACTS.

ACTS was shown to complicate 4.3% of distal radius fractures with a preponderance for females, similar to the results reported by Dyer et al [16]. All of the patients presented with paraesthesia in any of the fingers within the median nerve distribution. There were 14% of patients who experienced pain on top of paraesthesia, which is not a classical symptom of CTS. However, intense paraesthesia may often be misinterpreted as pain [28] and it can be difficult to distinguish between pain caused by the fracture and true carpal tunnel syndrome pain. In our series, signs of advanced disease including wasting of the thenar eminence and reduced two-point discrimination only presented after 5 weeks. This suggests that in the acute setting, the diagnosis of ACTS remains clinical and almost exclusive to paraesthesia in the median nerve distribution.

Documented risk factors in the literature that predispose to idiopathic CTS include diabetes, thyroid disease, pregnancy, BMI, smoking status, alcohol excess, SIMD and occupation [6, 7, 29–31]. However, no significant difference was demonstrated for these parameters in our study. Thyroid disease showed a trend towards the development of ACTS, and it is a known risk factor for idiopathic CTS. Patients with known thyroid disease should be counselled to recognise the symptoms and signs of developing CTS.

Although none of the ‘intrinsic’ factors seem to predispose patients to carpal tunnel syndrome, it appears that the fracture configuration does. Patients who sustained an AO-OTA type C fracture were more likely to experience ACTS. The results corroborate the findings from other studies, which showed that ACTS was more common in AO-OTA type C fractures [16]. However, no association was found with the mechanism of injury. The median nerve passes under the flexor retinaculum very close to the articular surface between the radius and the carpal bones and any injury involving the articular surface is more likely to cause median neuropathy. Patients with an AO-OTA type C fracture should undergo careful assessment of median nerve function during the acute presentation to ensure early intervention and prevent the morbidity associated with neglected carpal tunnel syndrome.

**Fig. 2:**
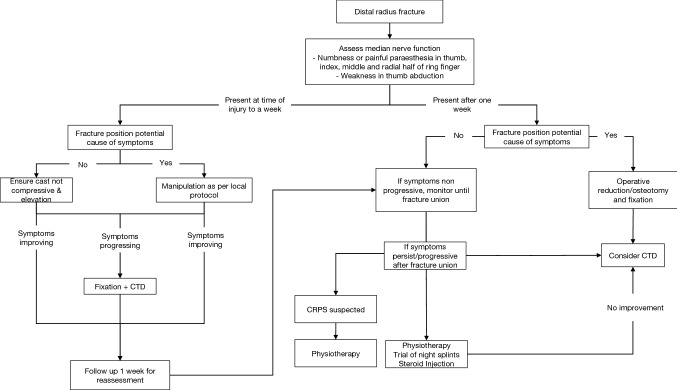
Suggested treatment algorithm for patients presenting with acute carpal tunnel syndrome following a fracture of the distal radius

There is no high-level evidence on the optimal management of ACTS following a distal radius fracture. Studies have recommended that patients presenting with signs and symptoms of ACTS and gross deformity in the wrist should be reduced and/or stabilised expeditiously [13, 32]. Patients who do not present with symptoms of ACTS should not undergo prophylactic CTD at the time of fixation [33]. If surgical fixation is carried out, a carpal tunnel decompression should be performed through a separate incision [13]. A number of patients in our cohort did not undergo CTD at the time of surgical fixation. The signs and symptoms of ACTS can result from a median nerve contusion and these cases can be treated with observation alone initially [32]. However, we would recommend a CTD should be performed if there is any uncertainty regarding the diagnosis or if the clinical picture has not improved or resolved. Kwasny et al [34, 35] reported that carpal tunnel syndrome after malunion of distal radius fractures can be treated with corrective osteotomy to reduce pressure of the transverse carpal ligament on the median nerve without a concomitant carpal tunnel decompression. Due to the acute nature of presentation, the published management of ACTS has been predominantly limited to case series and based on traditional teaching. In Fig. 2, the authors present a suggested treatment algorithm for these patients.

The main strength of our study is that it represents a large series of patients from a well-defined population that were prospectively recorded. Despite this, studies including a larger sample size are likely required to further investigate these associations. The primary limitation of this study is with the definition of ACTS. We believe that patients who have median nerve contusion cannot be reliably distinguished from progressive ACTS in the acute setting and have used the definition of paraesthesia in any of the fingers within the median nerve distribution as a diagnosis of ACTS. The retrospective nature of the study also meant that information including the exact onset of symptoms, nature of symptoms and two-point discrimination test were not recorded or retrievable. We have excluded cases of chronic carpal tunnel syndrome in our study. Although patients with chronic carpal tunnel syndrome may be more susceptible to acute exacerbations of their condition following a distal radius fracture, we believe that including patients with chronic CTS represents a confounding factor for development of ACTS secondary to a distal radius fracture. We also acknowledge that the treatment decision in our series was at the discretion of the treating surgeon, which it a clear limitation of retrospective work such as this and limits any firm conclusions regarding the optimal management algorithm for these patients. Future studies should incorporate prospective outcome data of patients who had carpal tunnel syndrome complicating a fracture of their distal radius. This could be used to investigate if this group of patients have inferior outcomes compared to those without CTS.
